# *Mycobacterium abscessus* and β-Lactams: Emerging Insights and Potential Opportunities

**DOI:** 10.3389/fmicb.2018.02273

**Published:** 2018-09-25

**Authors:** Elizabeth Story-Roller, Emily C. Maggioncalda, Keira A. Cohen, Gyanu Lamichhane

**Affiliations:** ^1^Division of Infectious Diseases, School of Medicine, Johns Hopkins University, Baltimore, MD, United States; ^2^Division of Pulmonary and Critical Care Medicine, School of Medicine, Johns Hopkins University, Baltimore, MD, United States

**Keywords:** *Mycobacterium abscessus*, β-lactams, peptidoglycan, LD-transpeptidase, β-lactamase inhibitor

## Abstract

β-lactams, the most widely used class of antibiotics, are well-tolerated, and their molecular mechanisms of action against many bacteria are well-documented. *Mycobacterium abscessus* (*Mab*) is a highly drug-resistant rapidly-growing nontuberculous mycobacteria (NTM). Only in recent years have we started to gain insight into the unique relationship between β-lactams and their targets in *Mab*. In this mini-review, we summarize recent findings that have begun to unravel the molecular basis for overall efficacy of β-lactams against *Mab* and discuss emerging evidence that indicates that we have yet to harness the full potential of this antibiotic class to treat *Mab* infections.

## Introduction

Although *Mycobacterium abscessus* (*Mab*) was first discovered in 1953 (Moore and Frerichs, 1953), it was only recently that genomic sequencing differentiated the *Mab* complex into three subspecies: *M. abscessus sensu stricto, M. abscessus* subsp. *bolletii*, and *M. abscessus* subsp. *massiliense* (Adekambi et al., 2004, 2006; Viana-Niero et al., 2008). These subspecies exhibit differential susceptibilities to certain antibiotics and differential clinical outcomes.

*Mab* can cause pulmonary disease in addition to skin and soft tissue infections, lymphadenitis, and disseminated disease. *Mab* is sometimes considered a respiratory colonizer; however, in the setting of immunosuppression or structural lung disease, such as cystic fibrosis (CF) and bronchiectasis, *Mab* can cause chronic pulmonary disease. In CF patients, *Mab* infections are often incurable and associated with rapid lung function decline (Griffith et al., 2007; Esther et al., 2010; Benwill and Wallace, 2014). The cure rate for *Mab* lung disease is only 30–50% (Jarand et al., 2011), with a recent review reporting sputum culture conversion rates as low as 25% with antibiotic treatment alone (Diel et al., 2017).

Poor treatment outcomes of *Mab* infection have been ascribed to both innate and acquired drug resistance. *Mab* is intrinsically resistant to multiple antibiotic classes which has been attributed to various factors (Brown-Elliott and Wallace, 2002; Nessar et al., 2012; van Ingen et al., 2012). Acquired resistance has further limited therapeutic options (Flume, 2016). Current treatment regimens are suboptimal, as they require several months of intravenous multidrug therapy with potentially cytotoxic antibiotics and produce poor outcomes (Wallace et al., 1985; Floto et al., 2016).

In this review, we will briefly summarize *Mab* treatment recommendations, discuss unique molecular targets of β-lactams in *Mab*, and highlight emerging insights into how β-lactams may be leveraged to treat individuals infected with *Mab*.

## Current *mab* treatment recommendations

The US Cystic Fibrosis Foundation and European Cystic Fibrosis Society recently developed consensus guidelines for management of *Mab* lung disease in CF patients (Floto et al., 2016). Similar to tuberculosis, *Mab* infection is treated with multidrug regimens divided into an intensive phase, followed by a continuation phase. Per recent guidelines, the intensive phase of *Mab* therapy should consist of an oral macrolide, combined with 3–12 weeks of intravenous amikacin, plus at least one of the following: intravenous cefoxitin, imipenem, or tigecycline (Floto et al., 2016). Guidelines for the continuation phase include a daily oral macrolide, inhaled amikacin, and two to three additional oral antibiotics, including minocycline, clofazimine, moxifloxacin, and linezolid.

Macrolides have historically been considered the backbone of treatment against *Mab*. They have relatively low toxicity, are orally bioavailable (Griffith et al., 2007; Floto et al., 2016), and exhibit consistent activity against *Mab in vitro* (Griffith et al., 2007). However, subspecies *abscessus* and *bolletii* harbor a functional *erm*(41) gene, which confers inducible macrolide resistance and can limit the effectiveness of this drug class. In contrast, subspecies *massiliense* carries a non-functional *erm*(41) gene (Nash et al., 2009), thus cannot exhibit inducible macrolide resistance and is associated with improved outcomes on macrolide-based regimens (Koh et al., 2011). Consequently, the CF guidelines recommend subspeciation of *Mab* complex, which many clinical laboratories are not equipped to perform routinely. Therefore, some CF centers prescribe initial treatment regimens comprised of intravenous amikacin plus either cefoxitin or imipenem, rather than a macrolide (Philley et al., 2016).

Cefoxitin and imipenem are currently the only two β-lactams included in the guidelines for treatment of *Mab* infections. This antibiotic class has been largely understudied against *Mab* and may be a potential untapped resource in combating this highly-resistant microbe.

## Mechanism of action of β-LACTAMS against *mab*

β-lactams are the most widely-used antibiotic class to treat bacterial infections (Hamad, 2010) and their safety and efficacy profiles have been well-established. There are five subclasses of β-lactams currently available in the clinical setting: penicillins, cephalosporins, monobactams, carbapenems, and penems. β-lactams have been studied extensively for treatment of drug-resistant *Mycobacterium tuberculosis* (*Mtb*) infections, which is summarized elsewhere (Story-Roller and Lamichhane, 2018). Certain β-lactam subclasses also exhibit activity against *Mab* (Lavollay et al., 2014; Kaushik et al., 2015; Lefebvre et al., 2016). While initial insights into the molecular mechanism of action of β-lactams against mycobacteria were gleaned largely from *Mtb*, recent studies have begun to elucidate the relationship between *Mab* and β-lactams (Lavollay et al., 2014; Lefebvre et al., 2016; Kumar et al., 2017a).

**Figure 1:**
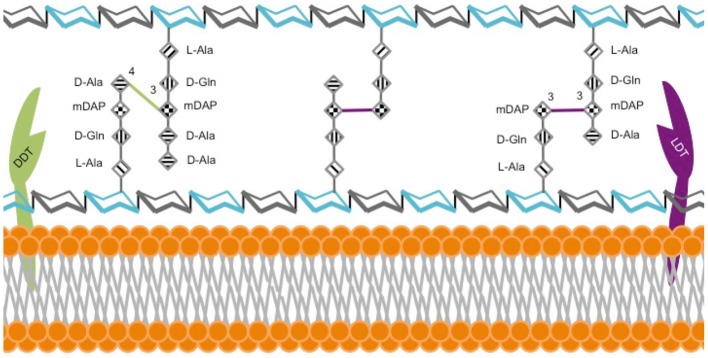
Model of *M. abscessus* peptidoglycan. The hexagonal structures depict sugars *N*-acetylglucosamine (gray) and *N*-acetylmuramic acid (cyan). L-alanine (L-Ala), D-glutamine (D-Gln), *meso-*diaminopimelic acid (m-DAP) and D-alanine (D-Ala).

β-lactams exert their activity by inhibiting synthesis of an essential component of the bacterial cell wall, the peptidoglycan (PG) (Hartmann et al., 1972). The building block of PG is a disaccharide with a stem peptide comprised of four or five amino acids; specifically *N*-acetyl-glucosamine-*N-*acetyl-muramic acid-l-alanyl-d-glutaminyl-*meso-*diaminopimelyl-d-alanyl-d-alanine in *Mab* (Lavollay et al., 2011). Polymerization of disaccharides by transglycosylases and stem peptides by transpeptidases produces a three-dimensional macromolecule, the PG (Figure 1).

The dominant model of PG architecture was largely established by studies using model organisms, such as *E. coli*. According to this historical model, the final step of PG synthesis is catalyzed by D,D-transpeptidases (DDT), also known as penicillin binding proteins, which link the 4th amino acid of one stem peptide to the 3rd amino acid of the adjacent stem peptide, thereby generating a 4 → 3-linked peptide network. However, as early as 1974, it became clear that the chemical architecture of mycobacterial PG, and therefore the enzymes necessary for its synthesis, were distinct from those described in the historical model. This study reported that stem peptides in the PG of *M. smegmatis, Mtb*, and *M. bovis* BCG were predominantly cross-linked with non-canonical linkages between the 3rd amino acid of one peptide and the 3rd amino acid of another (Wietzerbin et al., 1974). That same year, this group also demonstrated that the enzyme, L,D-transpeptidase (LDT), generated these 3 → 3 linkages in *Streptococcus faecalis* (Coyette et al., 1974).

The first direct evidence demonstrating that stem peptides in *Mab* PG are predominantly cross-linked by 3 → 3 linkages was reported in 2011 (Lavollay et al., 2011). Subsequently, five putative LDTs, Ldt_Mab1−5_, were identified in *Mab* (Mattoo et al., 2017) and the first crystal structure of one of these enzymes, Ldt_Mab2_, was described (Kumar et al., 2017a). These studies confirmed that *Mab* utilizes both LDTs and DDTs to generate 3 → 3 and 4 → 3 linkages between stem peptides, respectively (Figure 1). The majority of linkages in *Mab* are 3 → 3, which suggests that LDTs are at least as important as DDTs for synthesis of its PG. Several studies report that genes involved in PG synthesis and remodeling are largely conserved across mycobacteria, implying a similar PG chemical composition, architecture, and metabolism (Sanders et al., 2014; Mattoo et al., 2017). A review of PG biosynthesis in *Mtb* by Pavelka et al. is recommended for further insight into mycobacterial PG biology (Pavelka et al., 2014).

## LDTs are preferentially inhibited by carbapenems and cephalosporins

β-lactams mimic the *C-*terminal end of the native stem peptide of PG, bind to the active site of transpeptidases, and irreversibly inhibit their enzymatic activity (Park and Strominger, 1957). As the historical model considered DDTs to be the only enzymes that synthesized PG, they were assumed to be the sole targets of β-lactams. The discovery of LDTs (Mainardi et al., 2005) prompted inquiry into whether β-lactams also interacted with this enzyme class. Subsequent studies have demonstrated that LDTs and DDTs of mycobacteria differ in their binding affinities to β-lactam subclasses and are consequently inhibited by different subclasses to varying degrees (Dubee et al., 2012; Kumar et al., 2017b). DDTs are effectively bound and inhibited by all β-lactam subclasses, whereas *Mab* LDTs are preferentially bound and inhibited by carbapenems and to a lesser extent by cephalosporins (Kumar et al., 2017a,b).

Although the crystal structures of LDTs of *Mab* bound to β-lactams are not yet available, several groups have reported crystal structures of LDTs of *Mtb* bound to carbapenems and penems (Kim et al., 2013; Li et al., 2013; Bianchet et al., 2017; Kumar et al., 2017b; Steiner et al., 2017). As LDTs and DDTs of *Mab* are differentially inhibited by β-lactam subclasses, comprehensive inhibition of PG synthesis will likely require simultaneous administration of multiple β-lactams belonging to different subclasses to optimally inhibit the two enzyme classes.

## Factors that determine potency of β-LACTAMS against *mab*

The major molecular factors limiting effectiveness of β-lactams against *Mab* are β-lactamase activity and the bacterial cell wall. Factors that commonly affect other antibiotic classes, including poor permeability of the cellular envelope, low affinity of antibiotic targets, drug efflux pumps, and chromosomally-encoded neutralizing enzymes, have been elegantly summarized elsewhere (Nessar et al., 2012; van Ingen et al., 2012).

### β-lactamases

The potent activity of the chromosomally-encoded β-lactamase, Bla_Mab_, is primarily responsible for poor efficacy of β-lactams against *Mab* (Soroka et al., 2014). β-lactamases hydrolyze the β-lactam ring, thereby inactivating these antibiotics (Kasik et al., 1971). Not only does Bla_Mab_ degrade several β-lactams with significantly higher efficiency than BlaC of *Mtb*, Bla_Mab_ is not effectively inhibited by common β-lactamase inhibitors (BLI) clavulanate, tazobactam, and sulbactam (Soroka et al., 2017); agents that inhibit BlaC of *Mtb* (Wang et al., 2006). The observation that these BLIs do not reduce the minimum inhibitory concentration (MIC) of β-lactams against *Mab* in a whole-cell assay (Kaushik et al., 2017) is additional confirmation that β-lactamase activity in *Mab* is more robust than in *Mtb*. Subspecies *massiliense* harbors an additional β-lactamase, Bla_Mmas_ (Ramirez et al., 2017).

Bla_Mab_ is inactivated by avibactam (Dubee et al., 2015a), a recently-developed BLI whose core chemical composition differs from older BLIs and lacks a β-lactam ring (Coleman, 2011). Observations that avibactam reduces the MIC of several β-lactams against *Mab* provides further validation of its efficacy against both the Bla_Mab_ protein and whole-cell *Mab* (Dubee et al., 2015a; Kaushik et al., 2017; Lefebvre et al., 2017). A recent study showed avibactam not only inhibits β-lactamases but also inhibits LDTs (Edoo et al., 2018). A recombinant *Mab* strain lacking *bla*_*Mab*_ exhibited increased sensitivity to β-lactams and was rendered susceptible to amoxicillin and ceftaroline (Lefebvre et al., 2016). This study also observed that β-lactams plus avibactam exhibited similar efficacy against the parental *Mab* strain as compared to each drug alone against Δ*bla*_*Mab*_, suggesting that avibactam fully inhibits Bla_Mab._ While Bla_Mab_ and Bla_Mmas_ hydrolyze penicillins and cephalosporins with similar efficacy, Bla_Mmas_ also exhibits mild carbapenemase activity, a potential concern as it suggests an evolutionary movement toward β-lactamases with extended spectra (Ramirez et al., 2017). This study also noted that Bla_Mmas_ is structurally similar to other acquired carbapenemases normally found in gram negative bacteria, such as KPC-2 and SFC-1. Avibactam activity against Bla_Mmas_ has not yet been assessed and further study is warranted.

### Cell wall

Mycobacteria possess an unusually thick cell wall composed of layers of complex hydrophobic molecules including fatty acids, mycolic acids, lipoproteins, glycopeptidolipids (GPL), and largely insoluble PG and arabinogalactan layers. Although poorly-understood in *Mab*, epigenetic factors generating differential levels of these molecules, especially GPLs, are associated with two distinct colony morphotypes—rough and smooth—within a clonal population. The rough morphotype tends to be associated with higher rates of antimicrobial resistance, including against β-lactams (Cangelosi et al., 1999; Greendyke and Byrd, 2008; Lavollay et al., 2014). Additionally, glycosylation of lipoproteins limits permeability of the cell wall to antibiotics that inhibit PG synthesis (Becker et al., 2017). Cell wall porins are also partially responsible for β-lactam resistance, as they allow transport of small hydrophilic molecules across the membrane, which interact with targets within the cytoplasm to potentially activate expression of drug resistance genes (Nguyen and Thompson, 2006; Nessar et al., 2012).

## Activity of β-LACTAMS against *mab*

We identified thirty-five studies with documented MIC ranges of β-lactams against clinical isolates of *Mab* globally (Table 1). These data serve to highlight the high degree of variability in observed MIC ranges among clinical isolates, even within each study, and this variability is partially why standardized treatment regimens against *Mab* are often not practical in the clinical setting. Imipenem and cefoxitin were the most commonly-tested β-lactams and nearly all studies included *Mab* strains that were resistant to these agents based on established MIC breakpoints (Woods et al., 2011). Only in four studies were all strains susceptible or intermediate to cefoxitin (Lee et al., 2012; Lavollay et al., 2014; Singh et al., 2014; Jeong et al., 2017). Two studies performed subspeciation and observed that all strains of subspecies *massiliense* and/or *bolletii* were either susceptible or intermediate to imipenem, whereas subspecies *abscessus* exhibited higher MICs to this drug (Lavollay et al., 2014; Singh et al., 2014). The reason for this is not currently known. Although seventeen studies also evaluated additional β-lactams, it is evident that this antibiotic class is largely understudied against *Mab*.

**Table 1:** MIC range (ug/mL) for β-lactam antibiotics against clinical isolates of *M. abscessus*.

**Description of *M. abscessus* clinical isolates**	**BIA**	**DOR**	**ERT**	**FAR**	**IPM**	**MEM**	**PAN**	**TEB**	**FEP**	**CMZ**	**FOX**	**CRO**	**AMC**	**References**
3 isolates from US (TX)					8–64						16–512			Woods et al., 2000
8 isolates from Japan					8–16	8–16	4–16				16–>32		>32	Ito et al., 2003
92 isolates from Taiwan					1–>64	8–>64					8–256			Yang et al., 2003
48 isolates from South Korea					1–64						16–128			Lee et al., 2007
167 isolates from Taiwan					< 0.5–>64					< 1–>32	< 2–>256			Huang et al., 2008
74 isolates from Korea					4–>16						< 16–64			Park et al., 2008
45 isolates from South Korea					2–64						4–128			Jeon et al., 2009
108 isolates from US (UT)		16–>32	32–>32		2–>32	32–>32								Chihara et al., 2010
40 isolates from Taiwan					1–256						16–256			Huang et al., 2010
3 isolates from India (Mumbai)					64						256	64	32	Set et al., 2010
37 isolates from US (TX)					4–>16									Brown-Elliott et al., 2012
86 clinical isolates from Japan (63 subsp. abscessus, 23 massiliense)					2–64 4–64									Harada et al., 2012
6 isolates from Taiwan					32–64				>32		32–64	>64	>64	Lee et al., 2012
177 isolates from UK					< 4–>16				>32		< 16–>128	< 8–>64	16–>32	Broda et al., 2013
43 isolates from France/Germany (15 subsp. abscessus, 14 massiliense, 14 bolletii)					< 4–16 < 4 < 4–8						8–16 8–32 8–32			Lavollay et al., 2014
143 isolates from Japan (90 subsp. abscessus, 53 bolletii)					2–64 1–64				16–>256 1–>256			>32 1–>32	8–>256 2–>256	Yoshida et al., 2013
70 isolates from China					1–64						16–128			Zhuo et al., 2013
30 isolates from Brazil (6 subsp. abscessus, 24 bolletii)											32–256 32–256	>64 >64	>64 >64	Candido et al., 2014
70 isolates from China (Beijing) (45 subsp. abscessus, 25 bolletii)					< 0.5–16 2–16						4–64 16–>256			Nie et al., 2014
14 isolates from Taiwan (4 subsp. abscessus, 10 bolletii)					16–32 16–64				32–>32 >32		32–128 32–64	>64 >64	>64 >64	Lee et al., 2014
67 isolates from France (42 subsp. abscessus, 21 massiliense, 24 bolletii)					4–32 4–8 4–16						2–64 2–8 2–64			Singh et al., 2014
38 isolates from Australia (20 subsp. abscessus, 18 massiliense)					8–>64 4–>64				>32 >32		32–>128 32–>128	64–>64 64–>64	64–>64 >64	Chua et al., 2015
3 isolates from US (MD)	6.25–12.5	3.12–6.25	>25	40–80	>25	>25	>80	40–80						Kaushik et al., 2015
55 isolates from China						< 4–>32				< 16–>64	< 16–>128		< 8–>32	Pang et al., 2015
313 isolates from Singapore					4–>64						4–>128	‘		Tang et al., 2015
22 isolates from China					0.5–256						8–256			Li et al., 2016
78 isolates from US (TX) (67 subsp. abscessus, 11 massiliense)			4 4		8–16 8–16	8–16 8–16								Brown-Elliott et al., 2016
30 isolates from Iran					1–256	1–64					2–256			Heidarieh et al., 2016
165 isolates from France					4–>64				16–>32		8–128	8–>64	2–>64	Mougari et al., 2016
13 isolates from Japan				>2	2–16	8–64		4–>4	32–>64	8–64				Hatakeyama et al., 2017
20 isolates from South Korea (10 subsp. abscessus, 10 massiliense)					2–32 8–64						16–32 16–64			Jeong et al., 2017
28 isolates from US (MD)	16–128	8–128	64–>256	64–256	4–32	8–128	32–256	128–>256						Kaushik et al., 2017
67 isolates from Taiwan (28 subsp. abscessus, 38 massiliense, 1 bolletii)					8–>64 4–>64 32						16–128 16–>128 64			Lee et al., 2017
28 isolates from US (MD)					4–>64				16–>32		16–>128	32–>64	32–>64	Schwartz et al., 2018
64 isolates from US (FL)					< 4–>16						< 16–>128		16–>32	Sfeir et al., 2018

*BIA, biapenem; DOR, doripenem; ERT, ertapenem; FAR, faropenem; IPM, imipenem; MEM, meropenem; PAN, panipenem; TEB, tebipenem; FEP, cefepime; CMZ, cefmetazole; FOX, cefoxitin; CRO, ceftriaxone; AMC, amoxicillin*.

## Further potentiation of β-LACTAMS against *mab* by BLIs

Several studies have investigated the ability of BLIs to potentiate β-lactams against *Mab*, both *in vitro* and *in vivo*. The combination of amoxicillin and avibactam effectively reduced abscess formation and prolonged survival of zebrafish infected with *Mab* reference strain ATCC 19977 compared to amoxicillin alone (Dubee et al., 2015a). A subsequent study found that a combination of imipenem and avibactam also prolonged zebrafish survival compared to imipenem alone (Lefebvre et al., 2017). Avibactam also decreases the MIC of ceftaroline against *Mab* (Dubee et al., 2015b). Combinations of carbapenems and avibactam against clinical isolates of *Mab* showed that avibactam reduced MICs to therapeutically-achievable levels (Kaushik et al., 2017). The greatest MIC reductions were noted with tebipenem, ertapenem, and panipenem; demonstrating that avibactam can successfully overcome β-lactamase activity and further suggests that carbapenems, especially those developed after imipenem, such as doripenem, biapenem and tebipenem, have untapped potential for use against *Mab* (Kaushik et al., 2017).

## Synergy studies with β-LACTAMS and other drugs

As combination regimens are essential for clinical management of *Mab* infections, several studies have evaluated antibiotic synergy against *Mab* with mixed results (Cremades et al., 2009; Shen et al., 2010; Bastian et al., 2011; Choi et al., 2012; van Ingen et al., 2012; Oh et al., 2014; Singh et al., 2014; Ferro et al., 2016; Mukherjee et al., 2017; Aziz et al., 2018; Pryjma et al., 2018; Schwartz et al., 2018; Zhang et al., 2018). *In vitro* studies have shown variable synergy of β-lactams in combination with other drugs. One study found no evidence of synergy among combinations of either imipenem or ertapenem with various other antibiotics (Cremades et al., 2009). However, another study reported high levels of synergy against *Mab* clinical isolates when clofazimine and amikacin were combined with several β-lactam subclasses (Schwartz et al., 2018). In a final study, rifampin combined with either doripenem or biapenem significantly reduced the MICs of both drugs to within therapeutic levels, compared with each carbapenem alone (Kaushik et al., 2015).

## Dual β-LACTAMS for *mab*

Given that different subclasses of β-lactams target distinct aspects of mycobacterial cell wall biosynthesis, *Mab* regimens that contain two β-lactams from different subclasses may have high efficacy in *Mab*. As mentioned above, mycobacterial DDTs are inhibited by all β-lactams, whereas LDTs are preferentially inhibited by carbapenems and cephalosporins (Kumar et al., 2017a,b). A combination of cefdinir and doripenem was observed to be synergistic against *Mab* 19977 (Kumar et al., 2017a), demonstrating that dual β-lactams have therapeutic potential against *Mab*. This promising finding warrants further investigation into the effects of dual β-lactams against clinical isolates of *Mab*, further potentiation with BLIs, and additional *in vivo* studies.

## Preclinical models and clinical trials

At least two groups have taken initiatives to develop animal models of *Mab* infection (Lerat et al., 2014; Obregon-Henao et al., 2015). Two studies have assessed efficacy of antibiotic treatment of mice infected with *Mab*, one of which included a β-lactam, cefoxitin. Lerat et al. assessed regimens containing clarithromycin, amikacin, or cefoxitin monotherapy vs. a three-drug combination in nude mice infected with *Mab* ATCC 19977. Cefoxitin monotherapy was equally effective as triple therapy, resulting in prolonged survival and reduced splenic bacillary loads compared to untreated controls (Lerat et al., 2014). Several clinical trials assessing efficacy of non-β-lactam antibiotics against NTMs have been undertaken (clinicaltrials.gov). To date, there are no published clinical trials that have specifically investigated β-lactams for the treatment of *Mab*; however, we are hopeful that an increasing awareness of β-lactams as viable treatment options may lead to clinical trials with this class in the future.

## Future directions and conclusions

There is a dearth of research exploring β-lactams as potential treatments for *Mab*. Given the increasing prevalence of highly drug-resistant *Mab* isolates leading to poor clinical outcomes, new therapeutic approaches are needed to adequately treat these infections. Given our understanding of the differential mechanisms of β-lactam subclasses, and the ability of certain BLIs to overcome β-lactamase activity, currently-available β-lactams are a largely untapped resource for *Mab* treatment. Of the β-lactam subclasses, carbapenems/penems have the greatest activity against *Mab*, followed by cephalosporins, then penicillins. As noted above (Kumar et al., 2017a), it is likely that combinations of different β-lactam subclasses are required to fully inhibit PG synthesis in *Mab*. This insight may partially explain why prior studies evaluating β-lactams individually have not shown significant efficacy against this microbe. Further investigation may identify novel treatments utilizing combinations of β-lactams that optimally inhibit the distinct enzymatic targets present in *Mab*.

Appropriate selection of companion BLIs is another area in which β-lactams can be potentiated for use against *Mab*. Several studies have demonstrated efficacy of the BLI avibactam in inhibiting Bla_Mab_ activity, which is a major factor contributing to the high MIC of most β-lactams against *Mab*. However, avibactam is currently only available as a coformulated combination with ceftazidime, which itself does not appear to have activity against *Mab* (Dubee et al., 2015a; Kaushik et al., 2017). If avibactam were to be made available as an individual formulation, this would significantly increase its clinical usefulness, as regimens could be tailored to combine it with any β-lactam shown to be effective against a particular microbe or strain. Recently, two novel carbapenem-BLI combinations have been developed. These are meropenem-vaborbactam, which was recently FDA-approved for use against gram-negative organisms, and imipenem-relebactam, which is currently in phase II clinical trials (Zhanel et al., 2018). There are no published studies assessing efficacy of these BLIs against *Mab*, but their coformulation with carbapenems may confer greater potential for clinical use and further studies with these drugs are certainly warranted. It is possible that β-lactam-BLI combinations will become integral to effective treatment of drug-resistant *Mab* in the future. Additional animal studies as well as clinical trials with this drug class will be essential for the development of novel treatment regimens with improved clinical outcomes. Furthermore, repurposing already FDA-approved β-lactams for use against *Mab* may allow for expedited clinical implementation of regimens that show promise in preclinical models.

## Author contributions

ES-R, EM, KC, and GL discussed relevant literature. ES-R and KC focused on clinical aspects of the literature and EM focused on the basic biology. ES-R, EM, KC, and GL wrote the manuscript.

### Conflict of interest statement

The authors declare that the research was conducted in the absence of any commercial or financial relationships that could be construed as a potential conflict of interest.

## References

[B1] Adekambi T. Berger P. Raoult D. Drancourt M. (2006). rpoB gene sequence-based characterization of emerging non-tuberculous mycobacteria with descriptions of *Mycobacterium bolletii* sp. nov., *Mycobacterium phocaicum* sp. nov. and *Mycobacterium aubagnense* sp. nov. Int. J. Syst. Evol. Microbiol. 56, 133–143. 10.1099/ijs.0.63969-0 16403878

[B2] Adekambi T. Reynaud-Gaubert M. Greub G. Gevaudan M. J. La Scola B. Raoult D.. (2004). Amoebal coculture of “*Mycobacterium massiliense*” sp. nov. from the sputum of a patient with hemoptoic pneumonia. J. Clin. Microbiol. 42, 5493–5501. 10.1128/JCM.42.12.5493-5501.2004 15583272 PMC535245

[B3] Aziz D. B. Teo J. W. P. Dartois V. Dick T. (2018). Teicoplanin – tigecycline combination shows synergy against *Mycobacterium abscessus*. Front. Microbiol. 9:932. 10.3389/fmicb.2018.00932 29867841 PMC5958212

[B4] Bastian S. Veziris N. Roux A. L. Brossier F. Gaillard J. L. Jarlier V.. (2011). Assessment of clarithromycin susceptibility in strains belonging to the Mycobacterium abscessus group by erm(41) and rrl sequencing. Antimicrob. Agents Chemother. 55, 775–781. 10.1128/AAC.00861-10 21135185 PMC3028756

[B5] Becker K. Haldimann K. Selchow P. Reinau L. M. Dal Molin M. Sander P. (2017). Lipoprotein glycosylation by protein-O-mannosyltransferase (MAB_1122c) contributes to low cell envelope permeability and antibiotic resistance of *Mycobacterium abscessus*. Front. Microbiol. 8:2123. 10.3389/fmicb.2017.02123 29163413 PMC5673659

[B6] Benwill J. L. Wallace R. J. Jr. (2014). *Mycobacterium abscessus*: challenges in diagnosis and treatment. Curr. Opin. Infect. Dis. 27, 506–510. 10.1097/QCO.0000000000000104 25268925

[B7] Bianchet M. A. Pan Y. H. Basta L. A. B. Saavedra H. Lloyd E. P. Lamichhane G. (2017). Structural insight into the inactivation of *Mycobacterium tuberculosis* non-classical transpeptidase LdtMt2 by biapenem and tebipenem. BMC Biochem. 18:8. 10.1186/s12858-017-0082-4 28545389 PMC5445500

[B8] Broda A. Jebbari H. Beaton K. Mitchell S. Drobniewski F. (2013). Comparative drug resistance of *Mycobacterium abscessus* and *M. chelonae* isolates from patients with and without cystic fibrosis in the United Kingdom. J. Clin. Microbiol. 51, 217–223. 10.1128/JCM.02260-12 23135941 PMC3536196

[B9] Brown-Elliott B. A. Killingley J. Vasireddy S. Bridge L. Wallace R. J. Jr. (2016). *In vitro* comparison of ertapenem, meropenem, and imipenem against isolates of rapidly growing mycobacteria and nocardia by use of broth microdilution and Etest. J. Clin. Microbiol. 54, 1586–1592. 10.1128/JCM.00298-16 27053677 PMC4879266

[B10] Brown-Elliott B. A. Mann L. B. Hail D. Whitney C. Wallace R. J. Jr. (2012). Antimicrobial susceptibility of nontuberculous mycobacteria from eye infections. Cornea 31, 900–906. 10.1097/ICO.0b013e31823f8bb9 22362004

[B11] Brown-Elliott B. A. Wallace R. J. Jr. (2002). Clinical and taxonomic status of pathogenic nonpigmented or late-pigmenting rapidly growing mycobacteria. Clin. Microbiol. Rev. 15, 716–746. 10.1128/CMR.15.4.716-746.2002 12364376 PMC126856

[B12] Candido P. H. Nunes Lde S. Marques E. A. Folescu T. W. Coelho F. S. De Moura V. C.. (2014). Multidrug-resistant nontuberculous mycobacteria isolated from cystic fibrosis patients. J. Clin. Microbiol. 52, 2990–2997. 10.1128/JCM.00549-14 24920766 PMC4136125

[B13] Cangelosi G. A. Palermo C. O. Laurent J. P. Hamlin A. M. Brabant W. H. (1999). Colony morphotypes on Congo red agar segregate along species and drug susceptibility lines in the *Mycobacterium avium*-intracellulare complex. Microbiology 145, 1317–1324. 10.1099/13500872-145-6-1317 10411258

[B14] Chihara S. Smith G. Petti C. A. (2010). Carbapenem susceptibility patterns for clinical isolates of *Mycobacterium abscessus* determined by the Etest method. J. Clin. Microbiol. 48, 579–580. 10.1128/JCM.01930-09 20018813 PMC2815599

[B15] Choi G. E. Min K. N. Won C. J. Jeon K. Shin S. J. Koh W. J. (2012). Activities of moxifloxacin in combination with macrolides against clinical isolates of *Mycobacterium abscessus* and *Mycobacterium massiliense*. Antimicrob. Agents Chemother. 56, 3549–3555. 10.1128/AAC.00685-12 22564831 PMC3393384

[B16] Chua K. Y. Bustamante A. Jelfs P. Chen S. C. Sintchenko V. (2015). Antibiotic susceptibility of diverse *Mycobacterium abscessus* complex strains in New South Wales, Australia. Pathology 47, 678–682. 10.1097/PAT.0000000000000327 26517625

[B17] Coleman K. (2011). Diazabicyclooctanes (DBOs): a potent new class of non-β-lactam β-lactamase inhibitors. Curr. Opin. Microbiol. 14, 550–555. 10.1016/j.mib.2011.07.026 21840248

[B18] Coyette J. Perkins H. R. Polacheck I. Shockman G. D. Ghuysen J. M. (1974). Membrane-bound DD-carboxypeptidase and LD-transpeptidase of *Streptococcus faecalis* ATCC 9790. Eur. J. Biochem. 44, 459–468. 10.1111/j.1432-1033.1974.tb03504.x 4209348

[B19] Cremades R. Santos A. Rodriguez J. C. Garcia-Pachon E. Ruiz M. Royo G. (2009). *Mycobacterium abscessus* from respiratory isolates: activities of drug combinations. J. Infect. Chemother. 15, 46–48. 10.1007/s10156-008-0651-Y 19280301

[B20] Diel R. Ringshausen F. Richter E. Welker L. Schmitz J. Nienhaus A. (2017). Microbiological and clinical outcomes of treating non-*Mycobacterium avium* complex nontuberculous mycobacterial pulmonary disease: a systematic review and meta-analysis. Chest 152, 120–142. 10.1016/j.chest.2017.04.166 28461147

[B21] Dubee V. Bernut A. Cortes M. Lesne T. Dorchene D. Lefebvre A. L.. (2015a). β-Lactamase inhibition by avibactam in *Mycobacterium abscessus*. J. Antimicrob. Chemother. 70, 1051–1058. 10.1093/jac/dku510 25525201

[B22] Dubee V. Soroka D. Cortes M. Lefebvre A. L. Gutmann L. Hugonnet J. E.. (2015b). Impact of β-lactamase inhibition on the activity of ceftaroline against *Mycobacterium tuberculosis* and *Mycobacterium abscessus*. Antimicrob. Agents Chemother. 59, 2938–2941. 10.1128/AAC.05080-14 25733512 PMC4394810

[B23] Dubee V. Triboulet S. Mainardi J. L. Etheve-Quelquejeu M. Gutmann L. Marie A. (2012). Inactivation of *Mycobacterium tuberculosis* L,D-transpeptidase Ldt_Mt1_ by carbapenems and cephalosporins. Antimicrob. Agents Chemother. 56, 4189–4195. 10.1128/AAC.00665-12 22615283 PMC3421625

[B24] Edoo Z. Iannazzo L. Compain F. Li De La Sierra Gallay I. Van Tilbeurgh H. Fonvielle M.. (2018). Synthesis of avibactam derivatives and activity on β-lactamases and peptidoglycan biosynthesis enzymes of mycobacteria. Chemistry 24, 8081–8086. 10.1002/chem.201800923 29601108

[B25] Esther C. R. Jr. Esserman D. A. Gilligan P. Kerr A. Noone P. G. (2010). Chronic *Mycobacterium abscessus* infection and lung function decline in cystic fibrosis. J. Cyst. Fibros. 9, 117–123. 10.1016/j.jcf.2009.12.001 20071249 PMC3837580

[B26] Ferro B. E. Srivastava S. Deshpande D. Pasipanodya J. G. Van Soolingen D. Mouton J. W.. (2016). Failure of the amikacin, cefoxitin, and clarithromycin combination regimen for treating pulmonary *Mycobacterium abscessus* infection. Antimicrob. Agents Chemother. 60, 6374–6376. 10.1128/AAC.00990-16 27458221 PMC5038227

[B27] Floto R. A. Olivier K. N. Saiman L. Daley C. L. Herrmann J. L. Nick J. A. (2016). US cystic fibrosis foundation and European Cystic Fibrosis Society consensus recommendations for the management of non-tuberculous mycobacteria in individuals with cystic fibrosis. Thorax 71, i1–22. 10.1136/thoraxjnl-2015-207360 26666259 PMC4717371

[B28] Flume P. A. (2016). US cystic fibrosis foundation and European Cystic Fibrosis Society consensus recommendations for the management of non-tuberculous mycobacteria in individuals with cystic fibrosis. J. Cyst. Fibros. 15, 139–140. 10.1016/S1569-1993(16)00018-7 27062882

[B29] Greendyke R. Byrd T. F. (2008). Differential antibiotic susceptibility of *Mycobacterium abscessus* variants in biofilms and macrophages compared to that of planktonic bacteria. Antimicrob. Agents Chemother. 52, 2019–2026. 10.1128/AAC.00986-07 18378709 PMC2415760

[B30] Griffith D. E. Aksamit T. Brown-Elliott B. A. Catanzaro A. Daley C. Gordin F.. (2007). An official ATS/IDSA statement: diagnosis, treatment, and prevention of nontuberculous mycobacterial diseases. Am. J. Respir. Crit. Care Med. 175, 367–416. 10.1164/rccm.200604-571ST 17277290

[B31] Hamad B. (2010). The antibiotics market. Nat. Rev. Drug Discov. 9, 675–676. 10.1038/nrd3267 20811374

[B32] Harada T. Akiyama Y. Kurashima A. Nagai H. Tsuyuguchi K. Fujii T.. (2012). Clinical and microbiological differences between *Mycobacterium abscessus* and *Mycobacterium massiliense* lung diseases. J. Clin. Microbiol. 50, 3556–3561. 10.1128/JCM.01175-12 22915613 PMC3486228

[B33] Hartmann R. Holtje J. V. Schwarz U. (1972). Targets of penicillin action in *Escherichia coli*. Nature 235, 426–429. 10.1038/235426a0 4553687

[B34] Hatakeyama S. Ohama Y. Okazaki M. Nukui Y. Moriya K. (2017). Antimicrobial susceptibility testing of rapidly growing mycobacteria isolated in Japan. BMC Infect. Dis. 17:197. 10.1186/s12879-017-2298-8 28270102 PMC5341166

[B35] Heidarieh P. Mirsaeidi M. Hashemzadeh M. Feizabadi M. M. Bostanabad S. Z. Nobar M. G.. (2016). *In vitro* antimicrobial susceptibility of nontuberculous mycobacteria in Iran. Microb. Drug Resist. 22, 172–178. 10.1089/mdr.2015.0134 26468990

[B36] Huang T. S. Lee S. S. Hsueh P. R. Tsai H. C. Chen Y. S. Wann S. R.. (2008). Antimicrobial resistance of rapidly growing mycobacteria in western Taiwan: SMART program 2002. J. Formos. Med. Assoc. 107, 281–287. 10.1016/S0929-6646(08)60088-1 18445541

[B37] Huang Y. C. Liu M. F. Shen G. H. Lin C. F. Kao C. C. Liu P. Y.. (2010). Clinical outcome of *Mycobacterium abscessus* infection and antimicrobial susceptibility testing. J. Microbiol. Immunol. Infect. 43, 401–406. 10.1016/S1684-1182(10)60063-1 21075707

[B38] Ito K. Hashimoto K. Ogata H. (2003). Activity of cephems and carbapenems against clinically isolated *Mycobacterium abscessus*. Kekkaku 78, 587–590. 10.11400/kekkaku1923.78.587 14577345

[B39] Jarand J. Levin A. Zhang L. Huitt G. Mitchell J. D. Daley C. L. (2011). Clinical and microbiologic outcomes in patients receiving treatment for *Mycobacterium abscessus* pulmonary disease. Clin. Infect. Dis. 52, 565–571. 10.1093/cid/ciq237 21292659

[B40] Jeon K. Kwon O. J. Lee N. Y. Kim B. J. Kook Y. H. Lee S. H.. (2009). Antibiotic treatment of *Mycobacterium abscessus* lung disease: a retrospective analysis of 65 patients. Am. J. Respir. Crit. Care Med. 180, 896–902. 10.1164/rccm.200905-0704OC 19661243

[B41] Jeong S. H. Kim S. Y. Huh H. J. Ki C. S. Lee N. Y. Kang C. I.. (2017). Mycobacteriological characteristics and treatment outcomes in extrapulmonary *Mycobacterium abscessus* complex infections. Int. J. Infect. Dis. 60, 49–56. 10.1016/j.ijid.2017.05.007 28522316

[B42] Kasik J. E. Severson C. D. Stearns N. A. Thompson J. S. (1971). Immunologic distinction of mycobacterial β-lactamase. J. Lab. Clin. Med. 78, 982. 5002266

[B43] Kaushik A. Gupta C. Fisher S. Story-Roller E. Galanis C. Parrish N.. (2017). Combinations of avibactam and carbapenems exhibit enhanced potencies against drug-resistant *Mycobacterium abscessus*. Fut. Microbiol. 12, 473–480. 10.2217/fmb-2016-0234 28326811 PMC5618940

[B44] Kaushik A. Makkar N. Pandey P. Parrish N. Singh U. Lamichhane G. (2015). Carbapenems and rifampin exhibit synergy against *Mycobacterium tuberculosis* and *Mycobacterium abscessus*. Antimicrob. Agents Chemother. 59, 6561–6567. 10.1128/AAC.01158-15 26259792 PMC4576034

[B45] Kim H. S. Kim J. Im H. N. Yoon J. Y. An D. R. Yoon H. J.. (2013). Structural basis for the inhibition of *Mycobacterium tuberculosis* L,D-transpeptidase by meropenem, a drug effective against extensively drug-resistant strains. Acta Crystallogr. D Biol. Crystallogr. 69, 420–431. 10.1107/S0907444912048998 23519417 PMC3605043

[B46] Koh W. J. Jeon K. Lee N. Y. Kim B. J. Kook Y. H. Lee S. H.. (2011). Clinical significance of differentiation of *Mycobacterium massiliense* from *Mycobacterium abscessus*. Am. J. Respir. Crit. Care Med. 183, 405–410. 10.1164/rccm.201003-0395OC 20833823

[B47] Kumar P. Chauhan V. Silva J. R. A. Lameira J. D'andrea F. B. Li S. G. (2017a). *Mycobacterium abscessus* L,D-transpeptidases are susceptible to inactivation by carbapenems and cephalosporins but not penicillins. Antimicrob. Agents Chemother. 61:e00866–17. 10.1128/AAC.00866-17 28760902 PMC5610527

[B48] Kumar P. Kaushik A. Lloyd E. P. Li S. G. Mattoo R. Ammerman N. C.. (2017b). Non-classical transpeptidases yield insight into new antibacterials. Nat. Chem. Biol. 13, 54–61. 10.1038/nchembio.2237 27820797 PMC5477059

[B49] Lavollay M. Dubee V. Heym B. Herrmann J. L. Gaillard J. L. Gutmann L.. (2014). *In vitro* activity of cefoxitin and imipenem against *Mycobacterium abscessus* complex. Clin. Microbiol. Infect. 20, O297–O300. 10.1111/1469-0691.12405 24112243

[B50] Lavollay M. Fourgeaud M. Herrmann J. L. Dubost L. Marie A. Gutmann L.. (2011). The peptidoglycan of *Mycobacterium abscessus* is predominantly cross-linked by L,D-transpeptidases. J. Bacteriol. 193, 778–782. 10.1128/JB.00606-10 21097619 PMC3021224

[B51] Lee M. C. Sun P. L. Wu T. L. Wang L. H. Yang C. H. Chung W. H.. (2017). Antimicrobial resistance in *Mycobacterium abscessus* complex isolated from patients with skin and soft tissue infections at a tertiary teaching hospital in Taiwan. J. Antimicrob. Chemother. 72, 2782–2786. 10.1093/jac/dkx212 29091186

[B52] Lee M. R. Cheng A. Lee Y. C. Yang C. Y. Lai C. C. Huang Y. T.. (2012). CNS infections caused by *Mycobacterium abscessus* complex: clinical features and antimicrobial susceptibilities of isolates. J. Antimicrob. Chemother. 67, 222–225. 10.1093/jac/dkr420 21980068

[B53] Lee M. R. Ko J. C. Liang S. K. Lee S. W. Yen D. H. Hsueh P. R. (2014). Bacteraemia caused by *Mycobacterium abscessus* subsp. *abscessus* and *M. abscessus* subsp. *bolletii*: clinical features and susceptibilities of the isolates. Int. J. Antimicrob. Agents 43, 438–441. 10.1016/j.ijantimicag.2014.02.007 24718088

[B54] Lee S. M. Kim J. Jeong J. Park Y. K. Bai G. H. Lee E. Y.. (2007). Evaluation of the broth microdilution method using 2,3-diphenyl-5-thienyl-(2)-tetrazolium chloride for rapidly growing mycobacteria susceptibility testing. J. Korean Med. Sci. 22, 784–790. 10.3346/jkms.2007.22.5.784 17982223 PMC2693841

[B55] Lefebvre A. L. Dubee V. Cortes M. Dorchene D. Arthur M. Mainardi J. L. (2016). Bactericidal and intracellular activity of β-lactams against *Mycobacterium abscessus*. J. Antimicrob. Chemother. 71, 1556–1563. 10.1093/jac/dkw022 26929268

[B56] Lefebvre A. L. Le Moigne V. Bernut A. Veckerle C. Compain F. Herrmann J. L.. (2017). Inhibition of the β-lactamase Bla_Mab_ by avibactam improves the *in vitro* and *in vivo* efficacy of imipenem against *Mycobacterium abscessus*. Antimicrob. Agents Chemother. 61:e02440–16. 10.1128/AAC.02440-16 28096155 PMC5365697

[B57] Lerat I. Cambau E. Roth Dit Bettoni R. Gaillard J. L. Jarlier V. Truffot C.. (2014). *In vivo* evaluation of antibiotic activity against *Mycobacterium abscessus*. J. Infect. Dis. 209, 905–912. 10.1093/infdis/jit614 24253289

[B58] Li W. J. Li D. F. Hu Y. L. Zhang X. E. Bi L. J. Wang D. C. (2013). Crystal structure of L,D-transpeptidase Ldt_Mt2_ in complex with meropenem reveals the mechanism of carbapenem against *Mycobacterium tuberculosis*. Cell Res. 23, 728–731. 10.1038/cr.2013.53 23588382 PMC3641605

[B59] Li Y. M. Tong X. L. Xu H. T. Ju Y. Cai M. Wang C. (2016). Prevalence and antimicrobial susceptibility of *Mycobacterium abscessus* in a General Hospital, China. Biomed. Environ. Sci. 29, 85–90. 10.3967/bes2016.009 27003165

[B60] Mainardi J. L. Fourgeaud M. Hugonnet J. E. Dubost L. Brouard J. P. Ouazzani J.. (2005). A novel peptidoglycan cross-linking enzyme for a β-lactam-resistant transpeptidation pathway. J. Biol. Chem. 280, 38146–38152. 10.1074/jbc.M507384200 16144833

[B61] Mattoo R. Lloyd E. P. Kaushik A. Kumar P. Brunelle J. L. Townsend C. A.. (2017). Ldt_Mav2_, a nonclassical transpeptidase and susceptibility of *Mycobacterium avium* to carbapenems. Fut. Microbiol. 12, 595–607. 10.2217/fmb-2016-0208 28555497 PMC5619143

[B62] Moore M. Frerichs J. B. (1953). An unusual acid-fast infection of the knee with subcutaneous, abscess-like lesions of the gluteal region; report of a case with a study of the organism, *Mycobacterium abscessus*. J. Invest. Dermatol. 20, 133–169. 10.1038/jid.1953.18 13035193

[B63] Mougari F. Amarsy R. Veziris N. Bastian S. Brossier F. Bercot B.. (2016). Standardized interpretation of antibiotic susceptibility testing and resistance genotyping for *Mycobacterium abscessus* with regard to subspecies and erm41 sequevar. J. Antimicrob. Chemother. 71, 2208–2212. 10.1093/jac/dkw130 27147307

[B64] Mukherjee D. Wu M. L. Teo J. W. P. Dick T. (2017). Vancomycin and clarithromycin show synergy against *Mycobacterium abscessus in vitro*. Antimicrob. Agents Chemother. 61:e01298–17. 10.1128/AAC.01298-17 28923867 PMC5700366

[B65] Nash K. A. Brown-Elliott B. A. Wallace R. J. Jr. (2009). A novel gene, erm(41), confers inducible macrolide resistance to clinical isolates of *Mycobacterium abscessus* but is absent from *Mycobacterium chelonae*. Antimicrob. Agents Chemother. 53, 1367–1376. 10.1128/AAC.01275-08 19171799 PMC2663066

[B66] Nessar R. Cambau E. Reyrat J. M. Murray A. Gicquel B. (2012). *Mycobacterium abscessus*: a new antibiotic nightmare. J. Antimicrob Chemother. 67, 810–818. 10.1093/jac/dkr578 22290346

[B67] Nguyen L. Thompson C. J. (2006). Foundations of antibiotic resistance in bacterial physiology: the mycobacterial paradigm. Trends Microbiol 14, 304–312. 10.1016/j.tim.2006.05.005 16759863

[B68] Nie W. Duan H. Huang H. Lu Y. Bi D. Chu N. (2014). Species identification of *Mycobacterium abscessus* subsp. *abscessus* and *Mycobacterium abscessus* subsp. *bolletii* using rpoB and hsp65, and susceptibility testing to eight antibiotics. Int. J. Infect. Dis. 25, 170–174. 10.1016/j.ijid.2014.02.014 24932856

[B69] Obregon-Henao A. Arnett K. A. Henao-Tamayo M. Massoudi L. Creissen E. Andries K.. (2015). Susceptibility of *Mycobacterium abscessus* to antimycobacterial drugs in preclinical models. Antimicrob. Agents Chemother. 59, 6904–6912. 10.1128/AAC.00459-15 26303795 PMC4604395

[B70] Oh C. T. Moon C. Park O. K. Kwon S. H. Jang J. (2014). Novel drug combination for *Mycobacterium abscessus* disease therapy identified in a Drosophila infection model. J. Antimicrob. Chemother. 69, 1599–1607. 10.1093/jac/dku024 24519481

[B71] Pang H. Li G. Zhao X. Liu H. Wan K. Yu P. (2015). Drug susceptibility testing of 31 antimicrobial agents on rapidly growing mycobacteria isolates from China. Biomed. Res. Int. 2015:419392 10.1155/2015/419392 26351633 PMC4550772

[B72] Park J. T. Strominger J. L. (1957). Mode of action of penicillin. Science 125, 99–101. 10.1126/science.125.3238.99 13390969

[B73] Park S. Kim S. Park E. M. Kim H. Kwon O. J. Chang C. L.. (2008). *In vitro* antimicrobial susceptibility of *Mycobacterium abscessus* in Korea. J. Korean Med. Sci. 23, 49–52. 10.3346/jkms.2008.23.1.49 18303198 PMC2526484

[B74] Pavelka M. S. Jr. Mahapatra S. Crick D. C. (2014). Genetics of peptidoglycan biosynthesis. Microbiol. Spectr. 2:MGM2-0034-2013. 10.1128/microbiolspec.MGM2-0034-2013 26104213

[B75] Philley J. V. Degroote M. A. Honda J. R. Chan M. M. Kasperbauer S. Walter N. D.. (2016). Treatment of non-tuberculous mycobacterial lung disease. Curr. Treat Options Infect. Dis. 8, 275–296. 10.1007/s40506-016-0086-4 28529461 PMC5436303

[B76] Pryjma M. Burian J. Thompson C. J. (2018). Rifabutin acts in synergy and is bactericidal with frontline *Mycobacterium abscessus* antibiotics clarithromycin and tigecycline, suggesting a potent treatment combination. Antimicrob. Agents Chemother. 62:e00283–18. 10.1128/AAC.00283-18 29760147 PMC6105836

[B77] Ramirez A. Ruggiero M. Aranaga C. Cataldi A. Gutkind G. De Waard J. H.. (2017). Biochemical characterization of β-lactamases from *Mycobacterium abscessus* complex and genetic environment of the β-lactamase-encoding gene. Microb. Drug Resist. 23, 294–300. 10.1089/mdr.2016.0047 27429159

[B78] Sanders A. N. Wright L. F. Pavelka M. S. Jr. (2014). Genetic characterization of mycobacterial L,D-transpeptidases. Microbiology 160, 1795–1806. 10.1099/mic.0.078980-0 24855140 PMC4117223

[B79] Schwartz M. Fisher S. Story-Roller E. Lamichhane G. Parrish N. (2018). Activities of dual combinations of antibiotics against multidrug-resistant nontuberculous mycobacteria recovered from patients with cystic fibrosis. Microb. Drug Resist. 10.1089/mdr.2017.0286. [Epub ahead of print]. 29368988 PMC6200022

[B80] Set R. Rokade S. Agrawal S. Shastri J. (2010). Antimicrobial susceptibility testing of rapidly growing mycobacteria by microdilution–experience of a tertiary care centre. Indian J. Med. Microbiol. 28, 48–50. 10.4103/0255-0857.58729 20061764

[B81] Sfeir M. Walsh M. Rosa R. Aragon L. Liu S. Y. Cleary T.. (2018). *Mycobacterium abscessus* complex infections: a retrospective cohort study. Open Forum Infect. Dis. 5:ofy022. 10.1093/ofid/ofy022 29450214 PMC5808791

[B82] Shen G. H. Wu B. D. Hu S. T. Lin C. F. Wu K. M. Chen J. H. (2010). High efficacy of clofazimine and its synergistic effect with amikacin against rapidly growing mycobacteria. Int. J. Antimicrob. Agents 35, 400–404. 10.1016/j.ijantimicag.2009.12.008 20138481

[B83] Singh S. Bouzinbi N. Chaturvedi V. Godreuil S. Kremer L. (2014). *In vitro* evaluation of a new drug combination against clinical isolates belonging to the *Mycobacterium abscessus* complex. Clin. Microbiol. Infect. 20, O1124–O1127. 10.1111/1469-0691.12780 25185732

[B84] Soroka D. Dubee V. Soulier-Escrihuela O. Cuinet G. Hugonnet J. E. Gutmann L.. (2014). Characterization of broad-spectrum *Mycobacterium abscessus* class A β-lactamase. J. Antimicrob. Chemother. 69, 691–696. 10.1093/jac/dkt410 24132992

[B85] Soroka D. Ourghanlian C. Compain F. Fichini M. Dubee V. Mainardi J. L.. (2017). Inhibition of β-lactamases of mycobacteria by avibactam and clavulanate. J. Antimicrob. Chemother. 72, 1081–1088. 10.1093/jac/dkw546 28039278

[B86] Steiner E. M. Schneider G. Schnell R. (2017). Binding and processing of β-lactam antibiotics by the transpeptidase Ldt_Mt2_ from *Mycobacterium tuberculosis*. FEBS J. 284, 725–741. 10.1111/febs.14010 28075068

[B87] Story-Roller E. Lamichhane G. (2018). Have we realized the full potential of β-lactams for treating drug-resistant TB? IUBMB Life 70, 881–888. 10.1002/iub.1875. 29934998 PMC6119476

[B88] Tang S. S. Lye D. C. Jureen R. Sng L. H. Hsu L. Y. (2015). Rapidly growing mycobacteria in Singapore, 2006–2011. Clin. Microbiol. Infect. 21, 236–241. 10.1016/j.cmi.2014.10.018 25658536

[B89] van Ingen J. Boeree M. J. Van Soolingen D. Mouton J. W. (2012). Resistance mechanisms and drug susceptibility testing of nontuberculous mycobacteria. Drug Resist. Updat 15, 149–161. 10.1016/j.drup.2012.04.001 22525524

[B90] Viana-Niero C. Lima K. V. Lopes M. L. Rabello M. C. Marsola L. R. Brilhante V. C.. (2008). Molecular characterization of *Mycobacterium massiliense* and *Mycobacterium bolletii* in isolates collected from outbreaks of infections after laparoscopic surgeries and cosmetic procedures. J. Clin. Microbiol. 46, 850–855. 10.1128/JCM.02052-07 18174307 PMC2268380

[B91] Wallace R. J. Jr. Swenson J. M. Silcox V. A. Bullen M. G. (1985). Treatment of nonpulmonary infections due to *Mycobacterium fortuitum* and *Mycobacterium chelonei* on the basis of *in vitro* susceptibilities. J. Infect. Dis. 152, 500–514. 10.1093/infdis/152.3.500 3875667

[B92] Wang F. Cassidy C. Sacchettini J. C. (2006). Crystal structure and activity studies of the *Mycobacterium tuberculosis* β-lactamase reveal its critical role in resistance to β-lactam antibiotics. Antimicrob. Agents Chemother. 50, 2762–2771. 10.1128/AAC.00320-06 16870770 PMC1538687

[B93] Wietzerbin J. Das B. C. Petit J. F. Lederer E. Leyh-Bouille M. Ghuysen J. M. (1974). Occurrence of D-alanyl-(D)-meso-diaminopimelic acid and meso-diaminopimelyl-meso-diaminopimelic acid interpeptide linkages in the peptidoglycan of Mycobacteria. Biochemistry 13, 3471–3476. 10.1021/bi00714a008 4210702

[B94] Woods G. L. B. A Brown-Elliot P. S. Conville E. P. Desmond G. S. Hall G. (2011). Susceptibility Testing of Mycobacteria, Nocardiae, and Other Aerobic Actinomycetes, 2nd Edn. Wayne, PA: Clinical and Laboratory Standards Institute. 31339680

[B95] Woods G. L. Bergmann J. S. Witebsky F. G. Fahle G. A. Boulet B. Plaunt M.. (2000). Multisite reproducibility of Etest for susceptibility testing of *Mycobacterium abscessus, Mycobacterium chelonae*, and *Mycobacterium fortuitum*. J. Clin. Microbiol. 38, 656–661. 10655363 10.1128/jcm.38.2.656-661.2000 PMC86169

[B96] Yang S. C. Hsueh P. R. Lai H. C. Teng L. J. Huang L. M. Chen J. M.. (2003). High prevalence of antimicrobial resistance in rapidly growing mycobacteria in Taiwan. Antimicrob. Agents Chemother. 47, 1958–1962. 10.1128/AAC.47.6.1958-1962.2003 12760874 PMC155839

[B97] Yoshida S. Tsuyuguchi K. Suzuki K. Tomita M. Okada M. Hayashi S.. (2013). Further isolation of *Mycobacterium abscessus* subsp. *abscessus* and subsp. *bolletii* in different regions of Japan and susceptibility of these isolates to antimicrobial agents. Int. J. Antimicrob. Agents 42, 226–231. 10.1016/j.ijantimicag.2013.04.029 23850022

[B98] Zhanel G. G. Lawrence C. K. Adam H. Schweizer F. Zelenitsky S. Zhanel M. (2018). Imipenem-relebactam and meropenem-vaborbactam: two novel carbapenem-β-lactamase inhibitor combinations. Drugs 78, 65–98. 10.1007/s40265-017-0851-9 29230684

[B99] Zhang Z. Lu J. Song Y. Pang Y. (2018). *In vitro* activity between linezolid and other antimicrobial agents against *Mycobacterium abscessus* complex. Diagn. Microbiol. Infect. Dis. 90, 31–34. 10.1016/j.diagmicrobio.2017.09.013 29089153

[B100] Zhuo F. L. Sun Z. G. Li C. Y. Liu Z. H. Cai L. Zhou C.. (2013). Clinical isolates of *Mycobacterium abscessus* in Guangzhou area most possibly from the environmental infection showed variable susceptibility. Chin. Med. J. (Engl). 126, 1878–1883. 23673103

